# Pilomatricoma of the thigh: a case report

**DOI:** 10.11604/pamj.2022.43.208.34861

**Published:** 2022-12-28

**Authors:** Rita Ait Benhamou, Maria Kharbouch, Hamza Tazi, Zineb Basri, Mohamed Amine Lakhdari, Oqbani Kenza, Mounia El Omari

**Affiliations:** 1 Department of Plastic and Reconstructive Surgery, Cheikh Khalifa Hospital, Mohammed VI University of Health Sciences, Casablanca, Morocco,; 2 Department of Pathology, Cheikh Khalifa Hospital, Mohamed VI University of Health Sciences, Casablanca, Morocco

**Keywords:** Pilomatricoma, pilomatrixoma, skin neoplasm, case report

## Abstract

Pilomatricoma, formerly known as calcifying epithelioma of Malherbe, is a rare, benign, annexic skin tumor developed from the cells of the pilar matrix. The cure without recurrence is the rule after complete surgical excision. Clinical diagnosis is challenging. Actually, differential diagnosis include malignant pilomatricoma or trichomatrical carcinoma with significant aggressive potential. However, the diagnosis of pilomatricoma must remain clinical and be confirmed histologically. We report the rare case of a pilomatricoma, in an unusual location in the thigh.

## Introduction

Pilomatricoma, formerly known as calcifying epithelioma of Malherbe, is a rare, benign, annexic skin tumor developed from the cells of the pilar matrix [1]. It occurs during the first two decades of life. The most frequent locations are the head and neck, and the reach of the limbs remains exceptional. Clinical diagnosis is difficult. Several differential diagnoses can be evoked including malignant pilomatricoma or trichomatrical carcinoma with significant aggressive potential [2]. The diagnosis of pilomatricoma must remain clinical and be confirmed histologically. Its prognosis is generally good. The cure without recurrence is the rule after complete surgical excision [1,2]. The purpose of this work is to report the case of a patient with a rare tumor, pilomatricoma, in an unusual location the thigh.

## Patient and observation

**Patient information:** a 27-year-old patient, with no significant pathological history, has been seen for swelling of the antero-external side of the right thigh for 15 months.

**Figure 1:**
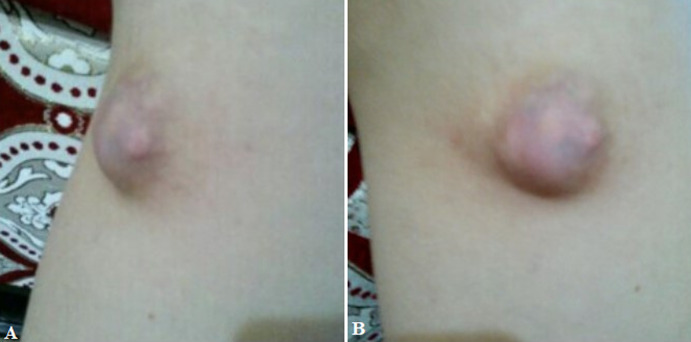
A, B) pre-operative pictures

**Clinical findings:** the clinical examination finds a subcutaneous swelling, hard, well limited, 3 cm long axis, adherent to the skin but mobile in relation to the deep plane (Figure 1). Loco regional examination found no palpable satellite lymphadenopathy.

**Figure 2:**
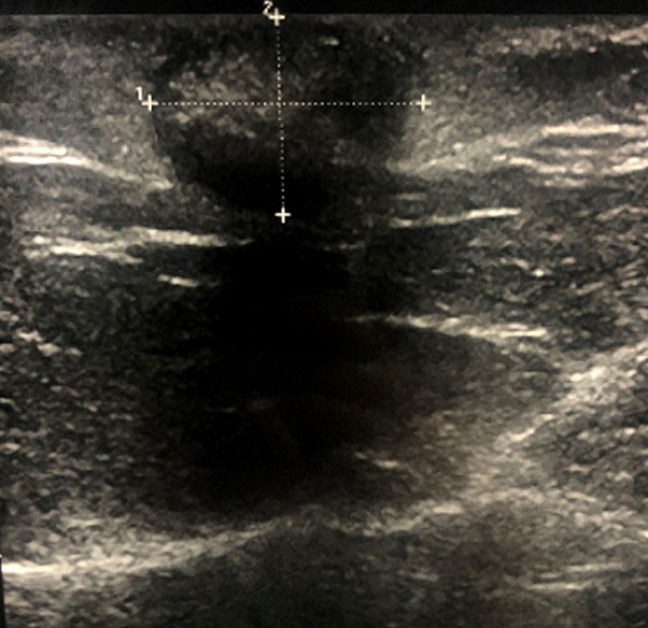
ultrasound showing very limited and homogeneous subcutaneous calcification

**Figure 3:**
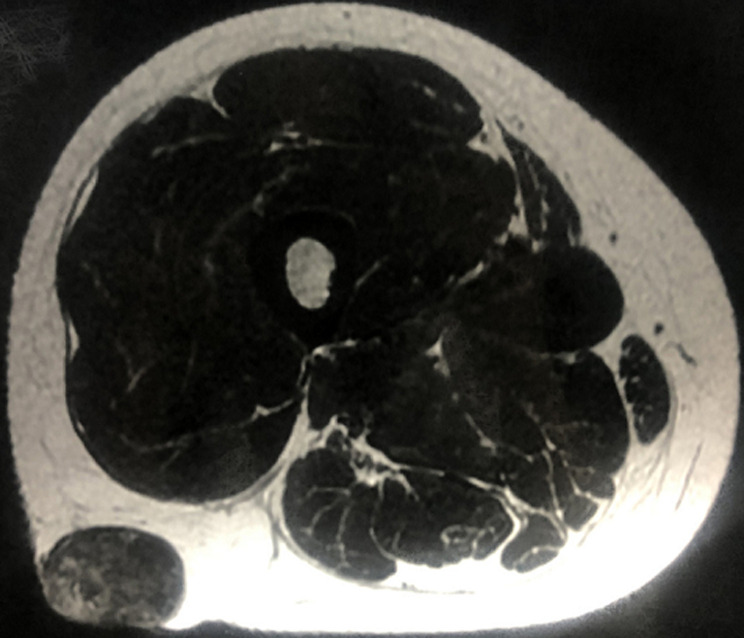
magnetic resonance imaging showing very limited and homogeneous subcutaneous calcification

**Diagnostic assessment:** ultrasound (Figure 2) and magnetic resonance imaging (MRI) (Figure 3) of the leg showed very limited calcification of the soft parts.

**Figure 4:**
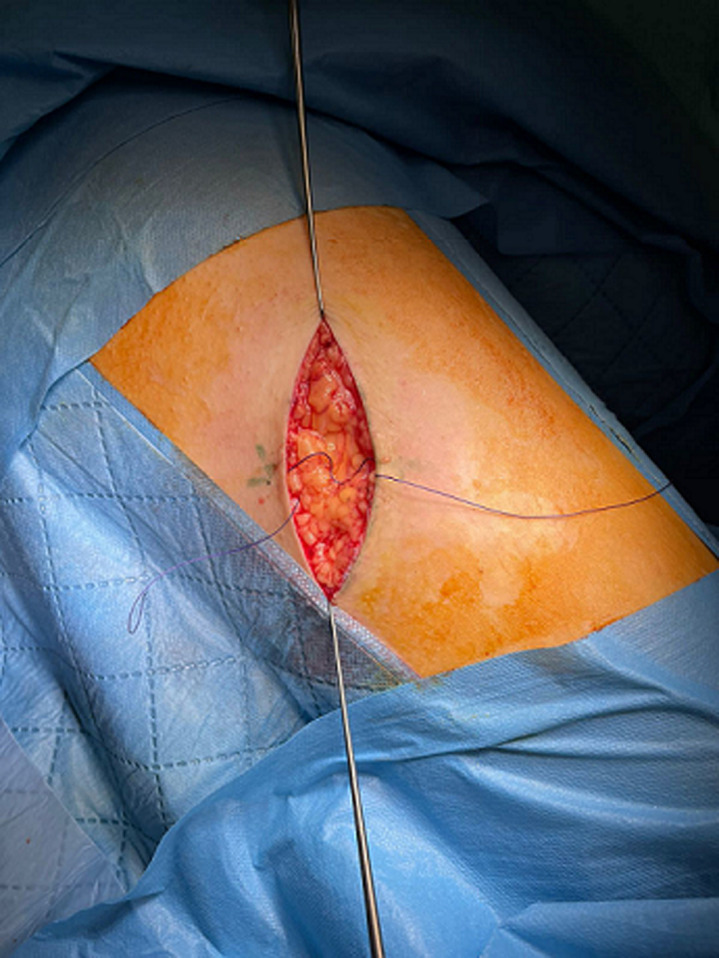
per-operative picture of the loss of substance after exeresis of the mass

**Figure 5:**
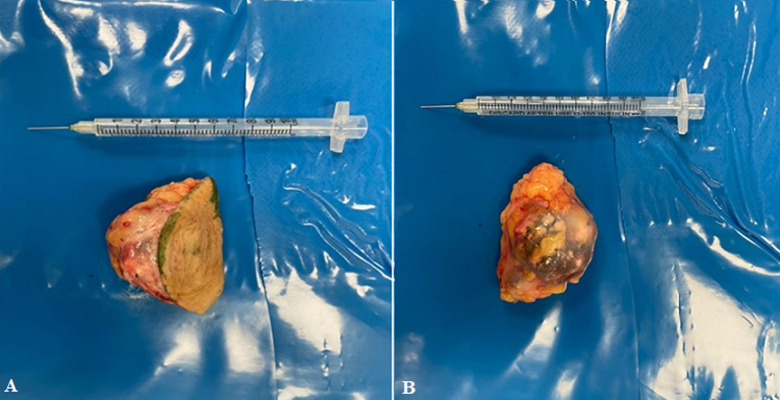
A, B) macroscopic appearance after total removal of pilomatricoma

**Figure 6:**
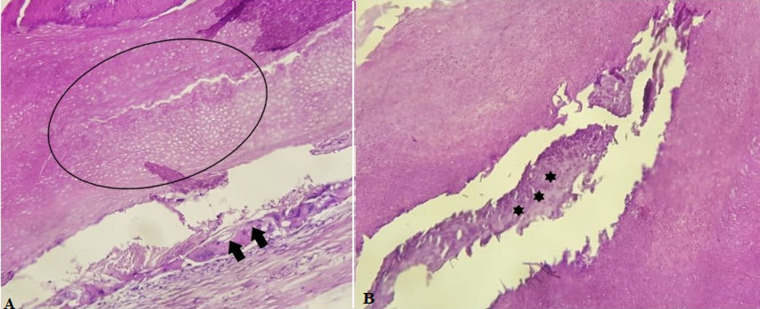
microscopic appearance showing Malherbe pilomatricoma (H&E x 40): A) mummified ghost cells (circle) surrounded by resorptive giant cells (arrows); B) calcifications (asterisks)

**Therapeutic interventions:** the patient benefited from surgical excision that removed a cutaneous spindle in front of the lesion under local anesthesia (Figure 4). The tumor nodule was encapsulated, indurated, measuring 3 cm long axis (Figure 5). The anatomopathological study noted the presence of mummified ghost cell layers surrounded by giant resorptive cells (Figure 6). Foci of calcifications were also observed (Figure 6).

**Follow-up and outcome of interventions:** no recurrence was noted at 6 months post-op.

**Informed consent:** the patient provided full consent after an oral explanation of our intention of publishing her case.

**Patient perspective:** the patient was satisfied with the treatment and the postoperative results.

## Discussion

Pilomatricome was first described by Wickens in 1858. But it is Malherbe and Chenantais in 1880, who defined the clinical and anatomopathological tumor [1]. Pilomatricoma mainly affects the child before the age of ten years with a female predominance (sex ratio of 1.5). It would also be more common in patients with Steinert myotonic dystrophy and in Gardner syndrome which associates rectocolic polyposis with extra digestive signs [1,2]. Pilomatricoma typically occurs as an asymptomatic subcutaneous nodule, round or oval, of hard or firm consistency, adhering to the superficial plane while being mobile to the deep planes [1,3]. The usual size is less than three centimeters, but cases of giant pilomatricoma exceeding five centimeters in diameter have been reported. It is generally unique, but some patients have already developed, simultaneously or successively, several pilomatricomas [3,4]. The usual locations are the neck and the head; rare are the cases that like our patient presented an isolated location at the level of the limbs. In most published cases, the difficulty of clinical diagnosis is based on the lack of knowledge of this tumor by some clinicians. Several differential diagnoses can be evoked such as a squamous and pilar cyst, subcutaneous fibroma, subcutaneous calcinosis but especially malignant pilomatricoma or trichomatrical carcinoma whose aggressive potential is important [3]. Standard radiography is only useful when faced with the suspicion of a pilomatricoma when it is significantly calcified [4,5]. The non-specific ultrasound diagnosis shows a very limited subcutaneous mass with a «target» appearance: an echogenic center and a hypoechoic thin circumference. The existence of a posterior shadow cone will indicate the presence of calcification. Magnetic resonance and computed tomography add little to the diagnosis. Histology is the only one able to confirm the diagnosis by identifying basaloid cells with or without ghost cells most often associated with calcifications [5]. The treatment of pilomatricoma consists of a complete surgical excision taking a skin spindle, especially if the lesion is adherent to the dermis. This is the reference treatment to avoid recurrence [1-5]. The prognosis of pilomatricoma is generally good; carcinomatous degeneration is still controversial to this day [5].

## Conclusion

Pilomatricoma is a rare skin tumor that should not be overlooked. This lesion is the most common of hair follicle tumors. The location at the level of the limbs remains exceptional. Its diagnosis is clinical, its confirmation is histological and its surgical treatment.
